# Using graph convolutional neural networks to learn a representation for glycans

**DOI:** 10.1016/j.celrep.2021.109251

**Published:** 2021-06-15

**Authors:** Rebekka Burkholz, John Quackenbush, Daniel Bojar

**Affiliations:** 1 Department of Biostatistics, Harvard School of Public Health, Boston, MA, USA; 2 Channing Division of Network Medicine, Department of Medicine, Brigham and Women’s Hospital and Harvard Medical School, Boston, MA, USA; 3 Department of Biostatistics and Computational Biology, Dana-Farber Cancer Institute, Boston, MA, USA; 4 Department of Chemistry and Molecular Biology, University of Gothenburg, Gothenburg, Sweden; 5 Wallenberg Centre for Molecular and Translational Medicine, University of Gothenburg, Gothenburg, Sweden

## Abstract

As the only nonlinear and the most diverse biological sequence, glycans offer substantial challenges for computational biology. These carbohydrates participate in nearly all biological processes—from protein folding to viral cell entry—yet are still not well understood. There are few computational methods to link glycan sequences to functions, and they do not fully leverage all available information about glycans. SweetNet is a graph convolutional neural network that uses graph representation learning to facilitate a computational understanding of glycobiology. SweetNet explicitly incorporates the nonlinear nature of glycans and establishes a framework to map any glycan sequence to a representation. We show that SweetNet outperforms other computational methods in predicting glycan properties on all reported tasks. More importantly, we show that glycan representations, learned by SweetNet, are predictive of organismal phenotypic and environmental properties. Finally, we use glycan-focused machine learning to predict viral glycan binding, which can be used to discover viral receptors.

## In brief

Burkholz et al. develop an analysis platform for glycans, using graph convolutional neural networks, that considers the branched nature of these carbohydrates. They demonstrate that glycan-focused machine learning can be employed for various purposes, such as to cluster species according to their glycomic similarity or to identify viral receptors.

## Graphical abstract

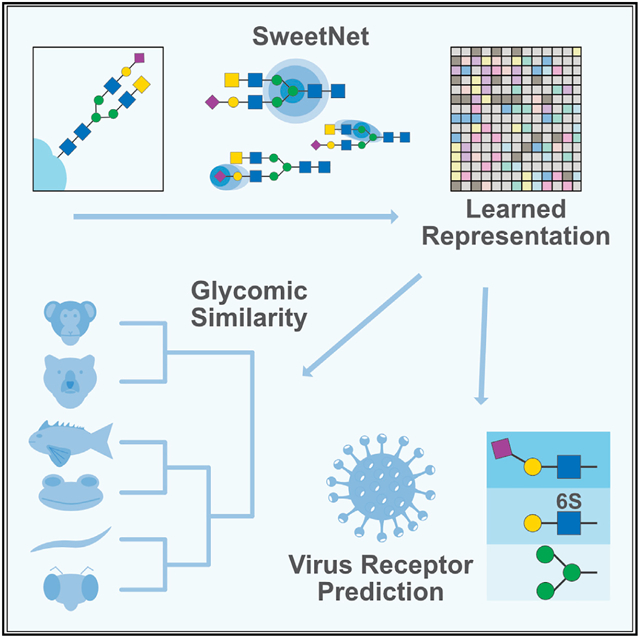

## INTRODUCTION

Glycans are complex carbohydrates and are a fundamental biological sequence that is found both as isolated entities as well as covalently bound to proteins, lipids, or other molecules (Varki, 2017). Because of their wide range of interactions, they are intricately involved in protein function (Dekkers et al., 2017; Solá and Griebenow, 2009), cellular function (Parker and Kohler, 2010; Zhao et al., 2008), and organismal function (Haltiwanger and Lowe, 2004; Stanley, 2016). As the only biological sequence with both a non-universal alphabet (consisting of monosaccharides) and nonlinear branching, glycans are highly complex biopolymers. This complexity is further compounded by the fact that glycans are created through a non-templated biosynthesis involving a stochastic interplay of multiple glycosyltransferases and glycosidases (Lairson et al., 2008), such as in the secretory pathway of eukaryotic cells that shapes the range of glycans on secreted proteins (Arigoni-Affolter et al., 2019).

Despite the important role that glycans inhabit in biology, the complexities in their composition and biosynthesis have slowed progress in the experimental and computational study of glycans. Computational approaches to analyzing glycans are mostly limited to counting the occurrence of curated sequence motifs and using this information as input for models predicting glycan properties (Bao et al., 2019; Coff et al., 2020). Recently, deep learning has been applied to the analysis of glycan sequences, creating glycan language models based on recurrent neural networks (Bojar et al., 2020a, 2020b, 2021). The glycan language model SweetTalk views glycans as a sequence of “glycowords” (subsequences that describe structural contexts of a glycan) and was used to predict the taxonomic class of glycans as well as their properties, such as immunogenicity or contribution to pathogenicity. While the usage of glycowords and additional data augmentation strategies in SweetTalk partly accounted for the nonlinear nature of glycan sequences, recurrent neural networks cannot fully capture the branched or tree-like architecture that is seen in most glycans. This implies that alternative model architectures that can fully integrate this nonlinearity should be able to extract more information from glycan sequences, thereby increasing prediction performance.

Advances in deep learning have produced a number of neural network architectures that are capable of analyzing graph- or tree-like structures (Henaff et al., 2015; Wu et al., 2021). These graph neural networks capitalize on the information contained in nodes and their connecting edges, as well as the contextual information contained in graph neighborhoods and modules, to predict properties of both individual nodes and entire graphs. One of the most useful methods in graph neural networks is message passing by convolutions, a procedure in which a node is described by a combination of the features of surrounding nodes (Henaff et al., 2015; Li and Cheng, 2020). Graph convolutional neural networks (GCNNs) have been used to great effect for studying social networks (Li et al., 2020) or epidemic forecasting (Kapoor et al., 2020) and have also been applied to proteins (Gligorijevic et al., 2019) and small-molecule drugs (Nguyen et al., 2020; Torng and Altman, 2019). In the latter, molecules are seen as molecular graphs, with atoms as nodes and bonds as edges. GCNNs also outperform widely used fingerprint-based methods in predicting small-molecule properties such as toxicity or solubility (Liu et al., 2019).

SweetNet is a deep learning method that we developed to take advantage of the flexible graph representation structure of GCNNs. SweetNet treats glycan sequences akin to molecular graphs and thereby accounts for their tree-like structures. Viewing monosaccharides and linkages as nodes and their connections as edges allows for the application of GCNNs to glycan sequences without any further manipulations or data augmentation. On a range of reported prediction tasks, we demonstrate that SweetNet yields considerably better prediction results than reported do glycan prediction models. We further demonstrate that the latent representations learned by SweetNet are more informative than those derived using other modeling methods. This improved performance is due to the representation of glycans as molecular graphs, a conclusion we also confirm by analyzing structural graph properties of glycans.

We demonstrate the value of SweetNet and the resulting glycan representations in two applications. First, we show that glycans contain information about phenotypic and environmental properties of their organisms that can be extracted via glycan representations. We use this phenomenon to identify phenotypic clusters in the plant order Fabales (dicotyledonous flowering plants that include the legumes), such as having pronounced seeds or fern-like leaves, that are clearly distinguished by their glycans. We further extend this to the kingdom Animalia, identifying clusters of animals inhabiting similar environmental niches (such as amphibians and fish). Our analyses highlight glycomic similarities in related groups and could enable the classification of phenotypically or environmentally similar organisms.

Finally, we show that SweetNet can be used to identify glycan receptors for viruses by presenting an additional glycan-focused prediction task: the prediction of the binding intensities between viral proteins and glycans. For this, we train a SweetNet-based model on a glycan array dataset probing interactions of influenza virus strains and glycans. We demonstrate that this model can then quantitatively predict the glycan-binding behavior of different influenza virus strains. Our model recapitulates known binding specificities of influenza virus, and we show that these predictions can be extended to other viruses, such as coronaviruses or rotaviruses. We add to these observations by identifying enriched binding motifs, such as complex motifs from human milk oligosaccharides for rotaviruses, indicating that our model can be used to rapidly identify glycan receptors for viruses. SweetNet thus represents a state of the art in glycan-focused machine learning and will enable future investigations into the important roles of glycans.

## RESULTS

### Developing a GCNN for glycans

The nonlinear branching structure of glycans, together with their diversity, has hitherto presented an obstacle to the development of machine learning models for glycobiology that fully capitalized on the rich information in glycan sequences. The use of a glycoword-based language model overcame some of these limitations, allowing for the prediction of glycan immunogenicity, pathogenicity, or taxonomic class (Bojar et al., 2020a, 2020b, 2021); data augmentation inspired by graph isomorphism further improved predictions (Bojar et al., 2021). This led us to consider whether the structure of glycans as graphs or trees could be better captured by neural network architectures specifically developed for modeling graphs. Therefore, we developed SweetNet, a GCNN that uses the connectivities and identities of monosaccharides and linkages in a glycan as input to predict glycan properties. While linkages might be intuitively interpreted as edges in a graph, we chose to characterize them as nodes. This decision was motivated by the prominence of short glycan motifs, such as the Tn antigen (“GalNAc(α1-”), which otherwise would have been precluded from our analyses.

To find an appropriate model architecture for a GCNN trained on glycan sequences, we chose the task of predicting which species a given glycan sequence came from as the task for building SweetNet. This multiclass classification, with 581 unique classes, represented one of the most challenging tasks for language models trained on glycan sequences, especially regarding rare classes, and thus offered a suitable challenge for identifying a better model architecture. We constructed neural networks with several different graph convolutional operators, including the simple graph convolutional operator (SGConv) (Wu et al., 2019), the GraphSAGE operator (Hamilton et al., 2018), and k-dimensional graph neural network operators (GraphConv) (Morris et al., 2020). All of the graph convolutional operators we considered outperformed language-model-based classifiers, which supported our hypothesis that graph-based models would be more appropriate for branching glycans. Among these, models based on GraphConv operators produced the best models (Table 1).

**Figure 1.**
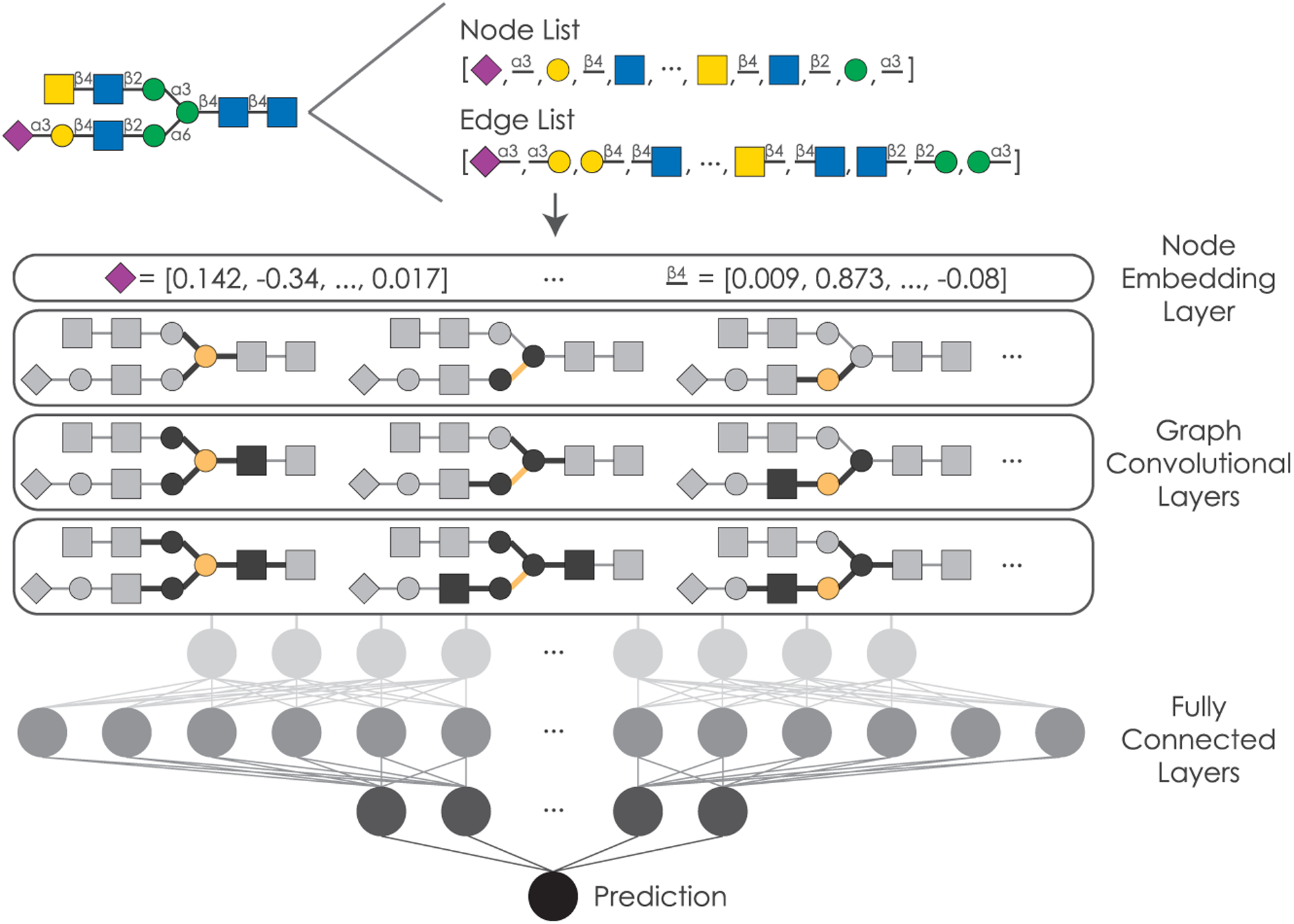
Schematic representation of the GCNN SweetNet for analyzing glycans Glycans are processed into a node list containing all occurring monosaccharides and linkages as well as a list of edge indices detailing the graph connectivity. This information is used as input for SweetNet by generating node features via a representation layer and then feeding the input through three graph convolutional layers. Subsequently, three fully connected layers, including a boom layer, use this information to generate a prediction.

We then sought to further enhance model performance by including a boom layer, which has been shown to enhance model performance in other contexts by escaping local minima (Merity, 2019), and observed a further increase in classification performance (Table 1). Hypothesizing that unsupervised pretraining on a larger set of glycans that included glycans without known species labels would further improve performance, we constructed a context prediction task (Hu et al., 2020) in which the model is used to predict the identity of a randomly chosen hidden node, given the connectivities and the other nodes in a glycan. We reasoned that this procedure should allow the model to learn more regularities and context effects from a larger set of diverse glycan sequences. While we were able to successfully pre-train our model, fine-tuning on the species prediction task did not further improve performance, suggesting that this context-dependent information was already incorporated during normal training. Overall, using SweetNet (Figure 1), we achieved an increase of nearly 8% in absolute accuracy for the challenging task of predicting the species of a glycan relative to the previous best method (SweetTalk).

### SweetNet outperforms alternative model architectures on all tasks

Next, we set out to demonstrate that a model architecture focusing on the inherent nature of glycans as molecular graphs is both robust and generalizable. For this, we tested SweetNet on all other prediction tasks that have been previously attempted with glycan-focused machine learning models, predicting higher taxonomic groups of a glycan, glycan immunogenicity, and association with pathogenicity. We found that SweetNet models outperformed all other methods for all prediction tasks (Table 2; Table S1). Compared to previous benchmarks (SweetTalk-based models), SweetNet-based models achieved absolute accuracy increases between 1% and 11% (average: 5.16%), depending on the prediction task. This made us confident that GCNNs are a more potent architecture for modeling glycan characteristics and functions.

Analogous to the species prediction task, we observed that context pre-training did not improve predictive performance, but including a boom layer did increase model performance in nearly all cases. Further, even with a boom layer, the performance of GraphSAGE operators was inferior to that of the GraphConv operator. We also observed that, when applied to the task of predicting the taxonomy of a glycan, SweetNet could be trained approximately 30% faster than the equivalent SweetTalk models. SweetNet was also considerably more data efficient than SweetTalk, surpassing SweetTalk performance even if only a third of the full dataset was used for training (Figure S1). These results confirmed our hypothesis that graph-based models are more efficient in extracting information from glycan sequences than alternative neural network architectures. Further, these data-efficient algorithms could allow prediction even given the relative scarcity of available glycan sequences, caused by the experimental difficulties of working with them.

As we anticipated, challenging prediction tasks with many classes and fewer datapoints per class, such as the prediction of species or genus, saw greater performance gains when using SweetNet than balanced, binary classifications. This is likely the result of a better match between the graph-like nature of glycans and the GCNN architecture, allowing the model to learn more associations and statistical dependencies to use for prediction. Further, the performance of SweetTalk-based models decreased with increased branching, while the performance of SweetNet-based models improved with increased branching (Figure S2), indicating that GCNNs such as SweetNet use structural properties unique to graphs, such as the number of branches or various connectivity statistics as features in the classification model.

We next generated a set of graph properties of the glycans in our dataset by calculating various connectivity statistics (Table S2; see STAR Methods for details) and trained a random forest classifier to predict the taxonomic kingdom of a glycan to evaluate whether models could extract information from purely structural features of glycans without their sequence. Our trained random forest model achieved a predictive accuracy of 61.4% on a separate test set—worse than the random forest model trained on sequence features (Table 2), yet substantially better than random predictions. This confirmed that graph properties by themselves are informative for predicting glycan properties, albeit possibly less so than sequence features.

Analyzing the feature importance of this model, we observed that the most important graph feature for a kingdom-level classification was the number of node types in a glycan (reflecting overall glycan sequence diversity; Figures S3A and S3B), which we also observed to be important for predicting the contribution of glycans to pathogenicity (Figure S3C), the immunogenicity of a glycan (Figure S3D), and the type of glycan (Figure S3E). To our surprise, we found that a smaller number of node types (meaning greater homogeneity in the glycan) predicted higher immunogenicity (Figures S3F and S3G); this result could be due to the presence of multiple binding sites for antibodies and the innate immune system. Other important features for predicting glycan immunogenicity and class, for instance, included aspects of the harmonic centrality, which is related to a graph’s compactness (Figures S3C–S3E).

### Glycan representations learned by SweetNet are more informative compared to alternative models

**Figure 2.**
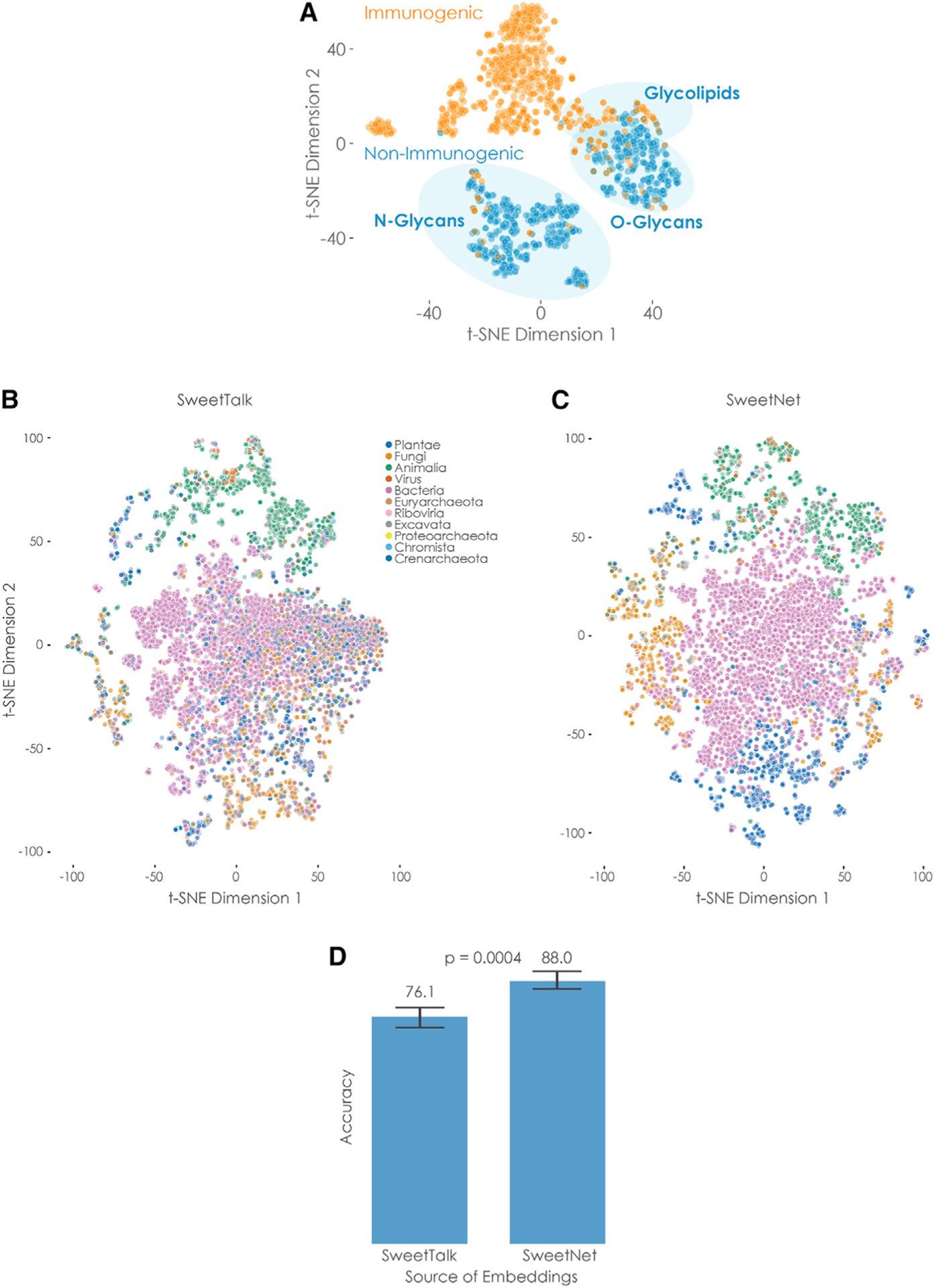
Comparison of glycan representations obtained by machine learning (A) Immunogenic glycan representations learned by SweetNet. Glycan representations for all glycans with immunogenic information were extracted from a trained SweetNet-based model and are shown via **t**-distributed stochastic neighbor embedding (t-SNE; van der Maaten and Hinton, 2008), colored by their immunogenicity label, and annotated by glycan classes. (B and C) Taxonomic glycan representations learned by SweetTalk and SweetNet. Glycan representations for all glycans with taxonomic information in our dataset were generated by SweetTalk (B) and SweetNet (C) trained on predicting the taxonomic genus a given glycan stemmed from. These representations are shown via t-SNE and are colored by their taxonomic kingdom. (D) Comparing information value of representations obtained by SweetTalk and SweetNet. Logistic regression models were trained on the representations obtained from the genus-level SweetTalk and SweetNet models in order to predict the taxonomic kingdom of a glycan. The achieved accuracy from representations from five training runs is shown here and was compared between models by a Welch’s t test (n per group = 5). See also Figures S3 and S4.

An additional advantage of deep learning models is that during training, representations of glycans are learned that can then be used for visualization and downstream prediction tasks. Reasoning that a model with superior prediction performance should have also learned more informative representations, we extracted glycan representations for some of the SweetNet models we trained. For this, we used glycan sequences as input for the trained model and extracted the results from the graph convolutional layers, directly prior to the fully connected part of the network. These representations can be visualized in two dimensions to identify clusters in the data. We demonstrated this with the example of the SweetNet-based model predicting glycan immunogenicity that resolved clear clusters of *N*- and *O*-linked glycans and glycolipids, respectively (Figure 2A). Further, the relative proximity of *O*-linked glycans and glycolipids to immunogenic bacterial glycans is consistent with phenomena of molecular mimicry (Bojar et al., 2021; Carlin et al., 2009), indicating that SweetNet incorporates biologically meaningful information.

As we observed the greatest performance increase of SweetNet relative to SweetTalk models in the taxonomic task of genus prediction, we compared glycan representations from SweetNet and SweetTalk models trained on this task (Bojar et al., 2021) (Figures 2B and 2C). Coloring glycans by kingdom allowed us to observe that SweetNet-based representations improved separation of glycans into taxonomic kingdoms, with clearer neighborhood separations; this can also be seen in the higher adjusted Rand index with K-means clustering kingdoms from SweetNet-based representations (0.203) than from SweetTalk-based representations (0.146).

To further quantify the information in these representations, we trained logistic regression models to predict the taxonomic kingdom of a glycan from its representation gained by genus-level SweetTalk or SweetNet, respectively. The SweetNet representations again demonstrated superior performance (Figure 2D; ~88% accuracy) compared to the model trained on SweetTalk representations (~76% accuracy), indicating the value of SweetNet representations for downstream analyses. The accuracy achieved by using representations from the genus-level SweetNet model even reached the level obtained by the kingdom-level SweetTalk model (Table 1), suggesting that cross-training for hierarchically related tasks could provide additional predictive power (Sarawagi et al., 2003). Additionally, representations learned by SweetNet recovered clusters of glycans with certain graph properties, such as a high number of node types or high average eigenvalues (Figure S4).

### Extracting phenotypic and environmental properties from glycan representations

**Figure 3.**
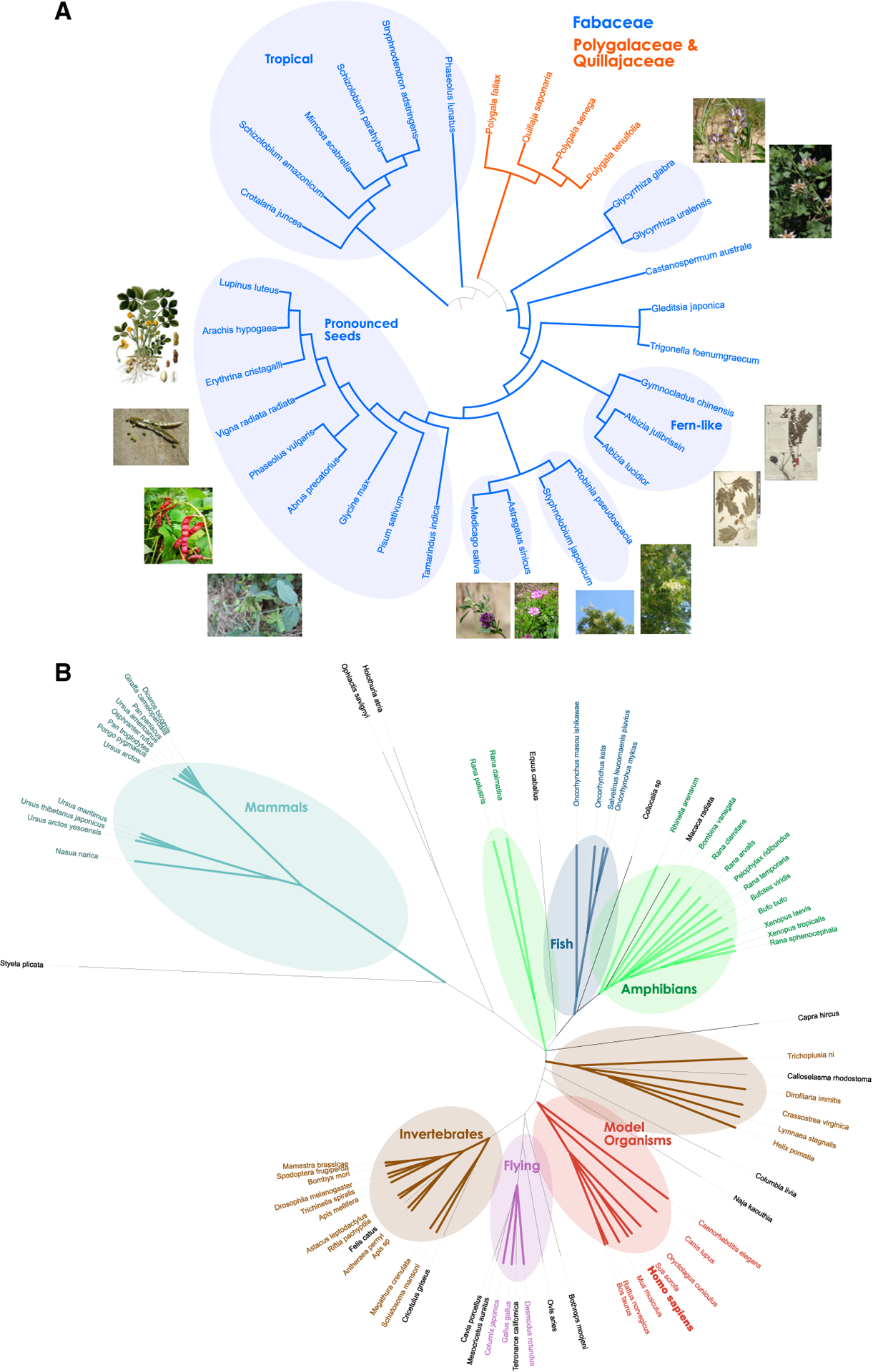
Glycan-based phylogenetic trees For all 30 species from the order Fabales with at least five glycans (A) or 93 species from the kingdom Animalia with at least two glycans in our dataset (B), we averaged their glycan representations from the species-level SweetNet model, constructed a cosine distance matrix among all species, and performed hierarchical clustering to obtain a dendrogram. The shown phylogenetic tree was drawn with the Interactive Tree of Life v5.5 software (Letunic and Bork, 2019). For (A), we colored species belonging to the taxonomic families Fabaceae and Polygalaceae/Quillajaceae. (A and B) We further annotated clusters enriched for certain groups of plants (A) or animals (B) that shared characteristics. The species *Homo sapiens* is depicted in bold. Images used for (A) stemmed from the public domain. Exceptions were from Creative Commons licenses requiring attribution and included *Astragalus sinicus* (https://commons.wikimedia.org/wiki/File:Chinese_milkvetch_Ziyunying.JPG), *Glycyrrhiza uralensis* (https://commons.wikimedia.org/wiki/File:Glycyrrhiza_uralensis_IMG_1086.jpg), and *Glycyrrhiza glabra* (https://commons.wikimedia.org/wiki/File:Glycyrrhiza_glabra_Y13.jpg).

Considering the rich glycan representations learned by SweetNet, we set out to improve on reported glycan-based phylogenies (Bojar et al., 2020a). Conveying phenotypic plasticity and covering every cellular surface, glycans are a major driver of evolution (Lauc et al., 2014) and mediate organismal properties more directly than DNA. Thus, a glycan-based phylogeny could offer insights into evolutionary histories and phenotypic similarity between species, complementary to that seen through DNA analysis. We constructed a proof-of-principle dendrogram of all species in the order Fabales with known glycans by averaging their glycan representations, constructing a cosine distance matrix, and performing hierarchical clustering. This revealed a clear clustering of taxonomic groups, with close association of species in the same genera and families that enabled us to establish a glycan-based phylogeny (Figure 3A).

In the dendrogram, we found clusters of plant species that share environmental and/or phenotypic similarities. This includes a cluster of plants occurring in tropical environments (South America and Africa); a cluster containing Fabaceae species that produce pronounced, mostly edible, seeds such as *Arachis hypogaea* (peanut) or *Glycine max* (soybean); and smaller clusters characterized by plants with similar leaf or flower phenotypes (Figure 3A). These results indicate that glycans carry considerable information about phenotypic and environmental characteristics of species and can be used to group species with shared properties and establish the notion of glycan-based relatedness, including the known effects of glycan-mediated phenotypic plasticity (Lauc et al., 2014).

We applied this method to all available species of the kingdom Animalia (Figure 3B) to draw a glycan-based tree of (animal) life. While differences in coverage prevented us from including all known species and distorted some relationships, we still were encouraged to see meaningful patterns emerge from this glycan-based phylogeny. We observed adjacent clusters for amphibians and fish, potentially reflecting their overlapping environmental range or their evolutionary history. We also find distinct clusters for flying animals (birds and bats), invertebrates, and mammals. It is interesting that *Homo sapiens* was absent from the cluster that included primates and instead was clustered with other well-studied model organisms, such as pigs, cows, mice, rats, rabbits, and dogs. In these organisms, *N*- and *O*-linked glycans, as well as glycolipids and free oligosaccharides, have been extensively analyzed, compared to perhaps more cursory or restricted analyses in more exotic organisms. This indicates that systematic factors, such as the degree of characterization, should be considered when interpreting these clustering results. The most closely associated species with humans in the model organism cluster, the pig *Sus scrofa*, is a prime candidate for xenotransplantation and is a major source of tissue for heart valve transplants (Burlak et al., 2013; Manji et al., 2015), supporting the close clustering of these two species we find here.

### Using SweetNet to explain virus-glycan binding

Most viruses bind to glycan receptors before, in some cases, transitioning to proteinaceous receptors during cell entry (Koehler et al., 2020; Thompson et al., 2019). The specificity and affinity of these glycan-binding events are essential for both virulence and host specificity, such as for influenza virus strains, which usually prefer either α2–3- or α2–6-linked sialic acids in their glycan receptors (Viswanathan et al., 2010). We constructed a model that, given a viral protein sequence and a glycan, could predict their interaction in the hopes of better understanding viral cell entry, developing methods to monitor emerging viral strains, and suggesting glycan-based antivirals.

Our model comprised a recurrent neural network analyzing the protein sequence, a module analyzing the glycan sequence (see below), and a fully connected part concatenating the results of both prior modules to predict the binding intensity of a protein-glycan pair. Using influenza virus and its glycan-binding protein hemagglutinin as a test case, we gathered 126,894 measured interactions between hemagglutinin variants with available sequences and glycans from the glycan array database of the Consortium for Functional Glycomics (Gao et al., 2019) (Table S3). Next, we determined which module for analyzing glycan sequences led to the highest prediction performance. For this, we tested three different modules: (1) a fully connected neural network that used the counts of mono-, di-, and trisaccharides of glycan sequences as input; (2) a SweetTalk-based glycan language model; and (3) a SweetNet-based GCNN that we introduced here. The SweetNet-based approach again yielded an improved performance over the currently available sequence motif-based and language-model-based approaches (Table 3; Table S1).

**Figure 4.**
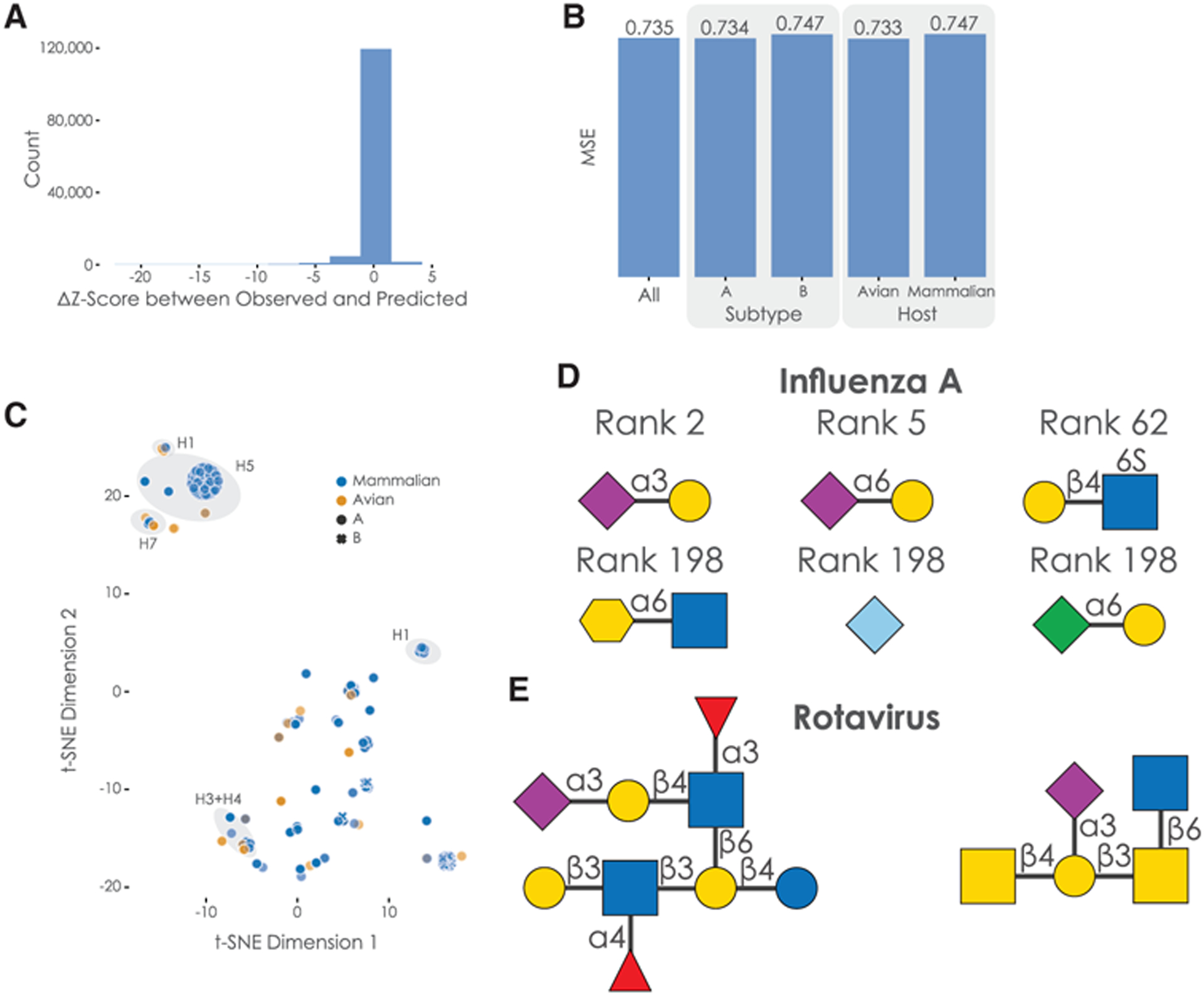
Characterizing the SweetNet-based model predicting virus-glycan binding (A) Distribution of residuals between predicted and observed *Z* scores. We calculated the difference between all observed and predicted *Z* scores of hemagglutinin-glycan binding interactions, shown here as a histogram. (B) Analyzing model performance on subgroups in the data. We obtained the mean square error (MSE) of our trained model for subgroups such as virus subtype or host organism in our data. (C) Hemagglutinin representations learned by the protein-analyzing module of the model. We obtained the representation learned by our protein-analysis module from the last state of our LSTM for all 339 unique protein sequences in our dataset. These representations are shown via t-SNE and colored/marked by their host organisms and virus subtype, respectively. Clusters of hemagglutinin subtypes are further annotated. (D) Examples of glycan motifs relevant for the prediction of hemagglutinin-glycan binding by SweetNet-based models. Some of the glycan motifs that were significantly relevant for prediction are shown in the symbol nomenclature for glycans (SNFG), together with their median rank across host species. (E) Examples of glycans with high predicted binding to rotavirus.

We then went on to ensure that this mean performance implied that most predictions would fall within this level of prediction error. For this, we collected all residuals between observed and predicted *Z* scores (Figure 4A), indeed observing that most residuals fall within the margin described by the mean prediction error. Further, the performance of our model remained stable for subgroups, such as virus subtypes or host organisms (Figure 4B).

Next, we analyzed the representations learned by the protein-analyzing long short-term memory (LSTM) module in our model, observing clustering according to the hemagglutinin subtype (Figure 4C). Additionally, while we did observe the separation of hemagglutinin from influenza A and B, the split between mammalian and avian hemagglutinin was less obvious, even though the classical view is that they differ considerably in their glycan-binding specificity. Supporting our findings, systematic binding studies have indeed identified several avian influenza hemagglutinin subtypes, such as H4 and H9, that exhibit binding properties similar to mammalian influenza hemagglutinin (Shelton et al., 2011).

We then identified glycan motifs that are important for the binding of hemagglutinin. Most approaches apply some form of subtree frequency mining to the glycan array data (Coff et al., 2020), identifying preferentially bound glycan fragments (Cholleti et al., 2012). However, we wanted to capitalize on the predictive nature of our trained model and used our 19,775 available glycan sequences—which are, for the most part, not covered by existing glycan arrays—as inputs to the trained model for each host species. Then, we analyzed the resulting binding predictions to ascertain which glycan motifs were most predictive for high-affinity binding across species (Figure 4D; see STAR Methods for further details). Assaying all 23,170 observed glycan motifs with lengths 1, 2, or 3 confirmed results achieved with standard methods, with Neu5Ac as the most important motif (median rank 1) and several known binding motifs such as Neu5Ac(α2–3)Gal (rank 2) or Neu5Ac(α2–6)Gal (rank 5; see Table S4 for all motifs). Published studies also suggest that sulfated glycan motifs may serve as binding motifs for influenza hemagglutinin (Ichimiya et al., 2014), which is consistent with our results (significant motifs with median ranks between 52 and 198; see Table S4).

We observed that the SweetNet-based model had learned relevant chemical information to predict hemagglutinin-glycan interactions by focusing on negatively charged motifs (containing carboxylates, sulfates, or phosphates) and sialic acids and structurally related monosaccharides in particular (Neu5Ac, Neu5Gc, Kdn, Kdo). This is especially encouraging, as monosaccharides such as Kdo (3-Deoxy-d-manno-oct-2-ulosonic acid), a bacterial analog of Kdn, were not present in the dataset used to train the model. This indicates that the model learns general features of glycans that are predictive of properties of “novel” glycan motifs. Although Kdo has, to our knowledge, not yet been proposed to bind influenza hemagglutinin, SweetNet suggests that it could be a target for influenza hemagglutinin.

We next wanted to test the generalizability of SweetNet for predicting other viral targets. For this, we trained a SweetNet model on a dataset combining the influenza virus glycan arrays with data from 83 additional glycan arrays that had been probed with a wide range of viruses (validation mean square error [MSE]: 0.784; Table S5). We then predicted the most important binding motifs for coronaviruses with this model and identified sialic acid motifs as well as sulfated glycan motifs (Table S6). These results are supported by reported binding motifs (Milewska et al., 2014) that naturally occur in glycosaminoglycans such as heparan sulfate. Next, we used the model to identify glycans with high predicted binding to rotaviruses, a common neonatal virus. Among the top 10 predicted glycans, we observed, next to expected sialic acid motifs, core 2 *O*-glycans (Figure 4E) that were indicated to bind rotaviruses (Pang et al., 2018). We additionally identified a glycan containing the Gal(β1–3)GlcNAc(β1–3)Gal(β1–4)Glc motif (Figure 4E; Table S7) that was previously described to be a potential decoy receptor from human milk that is bound by rotaviruses (Yu et al., 2014). Crucially, this glycan was not part of our training dataset, and the authors of this previous study had to use a specialized array made from human milk oligosaccharides to discover this binding motif. This demonstrates that SweetNet-based models can make useful predictions for unobserved glycans and positions our virus-glycan binding model to rapidly make predictions of bound glycan receptors for emerging virus variants.

## DISCUSSION

We tend to think of nucleic acids as the most important biological molecules, because they store the genetic code and facilitate protein synthesis, or proteins themselves because of their roles in mediating cellular processes and functions and serving in structural roles. However, cells are supported by a vast network of interacting biological molecules that also includes lipids and complex carbohydrates that serve in essential functional, metabolic, or structural capacities. Glycans represent a unique class of biological molecules in that they have nonlinear, branching structures that allow them to carry out a wide range of functions, encompassing protein folding and degradation; stress response; cell-cell interactions; cell migration patterns; self-/non-self-discrimination; and microbiome development, composition, and health (Varki, 2017).

Not surprisingly, glycans differ between species and change in response to environmental perturbations, and so they have the potential to allow us to understand genetic and environmental interactions (Lauc et al., 2014; Springer and Gagneux, 2016). Ideally, one would like to use glycan sequences to gain insights into phenotypic and environmental properties and to predict processes mediated by glycans such as viral infection. However, such applications are still rare, which may be due to the complex structure of glycans and the importance of these structures in determining glycan function.

GCNNs are a machine learning method that performs convolutional methods on the input graph itself (Henaff et al., 2015; Wu et al., 2021) with structure and features unchanged, rather than creating a lower-dimensional representation. Since the graph retains its original form, the relational inductive bias that is possible is much stronger. Given that glycans can be represented as complex graphs, we believed that GCNNs represented an ideal tool for applications involving glycan-based classification.

SweetNet is a GCNN implementation that fully leverages the tree-like structure of branched glycans. SweetNet-based models of glycans can be trained faster and are considerably more data efficient than models using other neural network architectures, and SweetNet outperforms these models in all of the tasks we analyzed. The data efficiency of SweetNet is an important metric because generating glycan data is not yet as easy or high-throughput as either DNA sequencing or proteomics, meaning this method can take advantage of the relatively sparse existing data available now, and its performance will improve as more data become available. To fully realize the potential of glycan-focused machine learning, it will be particularly crucial to gather more data from non-standard organisms as well as glycan classes beyond commonly investigated *N*-linked glycans.

In using glycan profiles from a wide range of species, we demonstrated that SweetNet can find taxonomic clusters that appear to group species based on both their evolutionary relationships and the ecological niche they inhabit, a finding consistent with the known genetic and environmental effects on glycan synthesis (Springer and Gagneux, 2016). Analyzed in this way, glycans could provide another window into similarities between species and their environments and may shed light on the role of glycans in phenotypic plasticity and evolution (Lauc et al., 2014).

Once more data become available, the GLYcosylation Metabolic Model of Enzyme Reactions (GLYMMER) software suite, used for connecting cellular glycomes with enzymatic capabilities (Bennun et al., 2013), could complement our analyses in the future. Mechanistic approaches such as GLYMMER elucidate biosynthetic pathways and can deepen our understanding of these cellular processes. Further, predicted glycan repertoires could also be used to cluster taxonomic groups. The value of our scalable approach with SweetNet lies in its capacity to rapidly analyze available glycan information from more than a thousand species without having to gather kinetic or specificity parameters for the involved enzymes. As these parameters become known for the species involved in this study, we envision that approaches such as GLYMMER can be used to extract more mechanistic insights from our analyses.

By choosing to consider both monosaccharides and linkages as nodes in our glycan graphs, we avoided a limitation of GCNNs that only glycans with a minimum length of at least two monosaccharides (disaccharides or larger) could be analyzed. Thus, SweetNet-based models can analyze important glycan structures such as the Tn antigen (“GalNAc(α1-”), which were inaccessible with previous language-model-based approaches, extending the potential applications for glycan-focused machine learning. Additionally, for applications involving these short glycans, the respective node representation learned by SweetNet could be used to extract information.

Ambiguities in nomenclature have spawned a plethora of different formats for describing glycans, yet most formats are either not human readable or insufficiently convey the branched structure of glycans. Depicting glycans as graphs circumvents these difficulties and offers the most promising nomenclature for predictive models in glycobiology. It also readily facilitates glycoengineering efforts (Kightlinger et al., 2020) by adding or removing nodes and the corresponding edges to a glycan graph and by querying trained models for the predicted properties of the proposed glycans. By using experimentally observed structure-activity relationships, such as the influence of N-glycans on antibody functionality and stability (Wada et al., 2019), models could be trained to predict glycans with improved properties, which could further be refined by removing undesired variants using the immunogenicity prediction model described here. Promising glycans could then be prioritized as design targets for antivirals or other purposes.

Further, existing analysis modalities of glycan substructures or subsequences (Bao et al., 2019) could readily be applied to the subset of glycans with high prediction scores to rationalize model predictions. Our trained models predicting virus-glycan binding could also be used to obtain relevant glycan-binding motifs to design glycan-based antivirals and additionally assess the potential of designed antiviral candidates by predicting their binding. As this represents only one of the areas of application for our platform, we envision a place in the design-build-test cycles of glycoengineering efforts for our SweetNet-based models.

Glycans have hitherto been neglected in most biological phenomena, at least in part because of the difficulties to work with and analyze glycans. The increasing number of applications in which glycan-focused machine learning has been shown to be feasible bodes well for finally lifting this analysis bottleneck and incorporating glycans into common analysis workflows. This is particularly emphasized by the development of model architectures and analysis platforms that are more data efficient, broadening the range of possible applications. Here, we advance both aspects, contributing applications for glycan-focused machine learning with our virus-glycan binding predictions and data-efficient models with our GCNN, SweetNet, that can already achieve state-of-the-art performance with small datasets. Our workflows are robust as well as rapid, and we envision the application of SweetNet-based models to many glycan-focused classification tasks.

## STAR★METHODS

### RESOURCE AVAILABILITY

#### Lead contact

Further information and requests for resources should be directed to and will be fulfilled by the Lead Contact, Daniel Bojar (daniel.bojar@gu.se)

#### Materials availability

This study did not generate new unique reagents.

#### Data and code availability

Data used for all analyses can be found in the supplementary tables. All code and trained models can be found at https://github.com/BojarLab/SweetNet

### METHOD DETAILS

#### Data processing

For comparing SweetNet to previously reported models, the data used in this study were largely from previous work (Bojar et al., 2021) and consisted of glycan sequences with their associated labels, such as taxonomic class, immunogenicity, or pathogenicity. For the model predicting viral glycan binding, we additionally obtained data from 587 glycan array screens from the Consortium for Functional Glycomics that measured the glycan binding behavior of hemagglutinin from various strains of influenza virus. For each array, we transformed the data to Z-scores. All data can be found in Table S3. For our expanded dataset, we also included Z score-transformed data from 83 arrays testing various viruses (Table S5).

For traditional machine learning models, we generated count variables that detailed the frequency of observed mono-, di-, and trisaccharide motifs in glycans and used these as input features. For glycan-based language models, we followed the data processing detailed previously (Bojar et al., 2021). Briefly, we extracted “glycowords” (trisaccharides in the IUPAC condensed bracket notation) from isomorphic variants of the bracket notation of a glycan and used these as input for a bidirectional recurrent neural network. For graph convolutional neural networks, we converted glycans from the bracket notation to graphs by generating a node list, in which every monosaccharide or linkage constituted a node, and a list of edge indices that detailed the graph connectivity.

For the model predicting viral glycan-binding, we selected a hemagglutinin core sequence that incorporated relevant binding loops (amino acid position 50 to 300) when the full sequence was available, to facilitate comparison to screens in which only partial sequences were available. Then, we label-encoded single amino acids in these sequences and used them as input for a recurrent neural network. This information was then combined with analyses of the corresponding glycan sequences by either 1) motif counting for a fully connected neural network, 2) a language model based on a recurrent neural network, or 3) a graph convolutional neural network.

#### Model training

All models were trained with PyTorch (Paszke et al., 2019) and PyTorch Geometric (Fey and Lenssen, 2019) on a single NVIDIA® Tesla® K80 GPU and the architecture as well as hyperparameters were optimized by minimizing the respective loss function via cross-validation of the training set. For all model applications, we used a random split into 80% training and 20% test data. As in previous work (Bojar et al., 2021), we used a stratified split for the taxonomic classifiers to ensure that all classes are split according to this ratio and also only consider classes with at least five known glycans. For the language models, all glycans were brought to the same length by padding. Language models were initialized using Xavier initialization (Glorot and Bengio, 2010) and GCNNs were initialized with a sparse initialization using a sparsity of 10%.

The final SweetNet model consisted of a 128-dimensional node representation layer followed by three iterations of graph convolutional layers, leaky ReLUs, Top-K pooling layers, and both global mean and global maximum pooling operations. The results from these three iterations were added and passed to a set of three fully connected layers interspersed with batch normalization layers, dropout layers, and leaky ReLUs as activation functions. For the final layer, we used a multi-sample dropout scheme (Inoue, 2019).

All models used a batch size of 32 for training and testing. The ADAM optimizer was used in all cases with a weight decay value of 0.001, together with a starting learning rate of 0.0005 that was decayed according to a cosine function over 80 epochs. Training proceeded for 100 epochs and was stopped early if the loss function did not decrease for at least 20 epochs. Depending on the application, we used binary cross-entropy, cross-entropy, or mean squared error loss functions.

#### Assessing the predictive value of structural graph features in glycans

We extracted 42 different graph features from each glycan to assess the predictive value of the graph representation from a structural point of view (Table S2). These features comprised the number of nodes, the number of different node types, the diameter of each graph (i.e., the maximum shortest path), its branching number (i.e., the number of nodes with 3 or more neighbors), the number of leaves (i.e., number of nodes with only one neighbor), and statistics on different centrality measures. Node and edge centrality measures assign a value to each node or edge in a graph to measure their respective importance. To define the same number of features for each graph with varying numbers of nodes, we always extracted the maximum value, the minimum value, the mean, and the variance across all nodes or edges. All considered centralities are implemented in the Python package NetworkX 2.5 (Hagberg et al., 2008). Specifically, we included degree centrality, betweenness centrality, flow centrality for nodes and edges, eigen centrality, closeness centrality, harmonic centrality, second order centrality, and load centrality. Usually, the degree (i.e., the number of neighbors a node has) plays a major role when comparing different graphs. As it also measures different aspects of branching, we included additional related features in our analysis: the degree assortativity, the number of nodes with at least four neighbors, and the maximum and mean number of leaves a node is connected to, which describes branching at potential binding sites. Furthermore, we added the maximum size of a k-core (comparing different values of k) and a corona to our set of features. These evaluate whether highly connected nodes tend to clique together. The most noteworthy features are the harmonic, flow, and second order centralities, which identify nodes as central that are close to most other nodes in the graph and are visited consistently by random walkers. Intuitively, our derived features are thus related to how compactly a graph is organized.

#### Identifying glycan motifs predictive for hemagglutinin-binding

All glycans in Table S8 were used as input for the trained SweetNet-based model predicting hemagglutinin-glycan binding. This was performed separately for hemagglutinin sequences from viruses of all host species (human, pig, dog, horse, bat, seal, duck, chicken, turkey, shorebird, gull). We then extracted all 23,170 glycan motifs of lengths 1, 2, and 3 from the glycan sequences that were observed in our dataset, as reported previously (Bojar et al., 2021). For each species, we then defined “predicted binding” as a predicted Z-score above 1.645, as suggested by related work on glycan array data (Cholleti et al., 2012). For each glycan motif, we performed a one-tailed Welch’s t test to ascertain whether glycans containing this motif were more prevalent among glycans predicted to bind. The resulting p values were subsequently corrected for multiple testing by a Holm-Sidák correction. To gauge the overall relevance of the significant glycan motifs, we then calculated the median rank of each significant motif across all host species.

### QUANTIFICATION AND STATISTICAL ANALYSIS

For statistical analysis, this study used Welch’s t tests with a Holm-Šidák correction for multiple testing correction. All experimental details can be found in the STAR Methods section and all statistical details of experiments can be found in the figure legends.

## Supplementary Material

Document S1. Figures S1–S4

Table S7. Binding predictions for glycans binding to rotavirus, related to Figure 4E

Table S6. Enriched glycan motifs for binding coronavirus, related to Figure 4

Table S8. All available glycan sequences for identifying enriched motifs, related to STAR Methods

Table S5. Glycan array data for various viruses, related to Figure 4

Table S4. Enriched glycan motifs for binding influenza virus hemagglutinin, related to Figure 4D

Table S3. Glycan array data for influenza virus hemagglutinin, related to Figure 4

Table S1. Model performances on glycan-focused tasks, related to Table 2

Table S2. Graph features of glycans in dataset, related to Figure 2

## Figures and Tables

**Table 1.** Selecting an architecture for a glycan-focused GCNN

Model	Crossentropy loss	Accuracy	Matthew’s correlation coefficient
Random forest	–	0.3025	0.2920
KNN	–	0.3146	0.3015
SweetTalk	3.9550	0.3651	0.3496
SGConv	2.5741	0.4000	0.3882
SAGEConv	2.5183	0.4097	0.3989
GraphConv	2.5173	0.4192	0.4078
GraphConv boom	2.3756^a^	0.4430^a^	0.4326^a^
GraphConv boom pretrain	2.5048	0.4278	0.4166

We trained several machine learning models (random forest, K-nearest neighbor), deep learning models such as the glycan-based language model SweetTalk, and GCNNs with different operators (SGConv, SAGEConv, GraphConv) for the prediction of which species a given glycan stemmed from. Mean values from five independent training runs (N = 5) for cross-entropy loss (except for random forest and KNN, which do not use this loss function), accuracy, and Matthew’s correlation coefficient on a separate test set are shown. The inclusion of a boom layer and context pretraining is indicated in the model column. See also Figures S1 and S2.

a The superior value for each metric.

**Table 2.** Comparison between different machine learning architectures to extract information from glycans

Application	KNN	RF	SweetTalk	GraphSAGE boom	Graph Conv	GraphConv boom (SweetNet)	GraphConv boom pretrain	Increase_SotA
Domain	0.845	0.868	0.931	0.929	0.933	0.942^a^	0.939	+1.09
Kingdom	0.819	0.834	0.895	0.902	0.903	0.910^a^	0.907	+1.42
Phylum	0.692	0.751	0.801	0.832	0.825	0.847^a^	0.841	+4.66
Class	0.574	0.650	0.715	0.741	0.743	0.757^a^	0.745	+4.16
Order	0.423	0.492	0.533	0.575	0.550	0.603^a^	0.582	+6.93
Family	0.345	0.387	0.466	0.520	0.518	0.554^a^	0.535	+8.78
Genus	0.335	0.326	0.385	0.468	0.463	0.496^a^	0.475	+11.09
Species	0.315	0.303	0.365	0.414	0.419	0.443^a^	0.428	+7.79
Immunogenicity	0.849	0.835	0.917	0.939	0.930	0.946^a^	0.942	+2.85
Pathogenicity	0.814	0.825	0.891	0.878	0.919^a^	0.897	0.904	+2.83

For a range of prediction tasks (Application), machine learning models (K-nearest neighbor classifier, random forest), deep-learning-based language models (SweetTalk), and GCNNs were tested. The choice of graph neural network operator (GraphSAGE, GraphConv) and the presence of a boom layer and pre-training are indicated. Mean accuracy values of five independent training runs (N = 5) for each model on a separate test set are given, and Matthew’s correlation coefficient values can be found in Table S1. Performance improvement relative to the previous state-of-the-art model (SweetTalk) is shown in absolute percent increase.

a The best value for each prediction task.

**Table 3.** Developing a model predicting viral glycan-binding behavior

Model	Train MSE	Test MSE
Fully connected	0.8508	0.8753
Language model (SweetTalk)	0.8253	0.8726
Graph model (SweetNet)	0.7455^a^	0.7352^a^

Models consisted of a recurrent neural network analyzing the protein sequences of viral hemagglutinin as well as either a fully connected neural network using the counts of mono-, di-, and trisaccharides as input (“Fully connected”); a SweetTalk-based glycan language model; or a SweetNet-based GCNN. All models were trained to predict *Z* score transformed glycan binding of hemagglutinin from various influenza virus strains. Average MSEs from five independent training runs (N = 5), from both the training and independent test set, are shown here.

a The superior value for each metric.

**Table:** KEY RESOURCES TABLE

REAGENT or RESOURCE	SOURCE	IDENTIFIER
Software and algorithms		
PyTorch	Paszke et al., 2019	https://github.com/pytorch/pytorch
Scikit-learn	Pedregosa et al., 2011	https://github.com/scikit-learn/scikit-learn
PyTorch Geometric	Fey and Lenssen, 2019	https://github.com/rusty1s/pytorch_geometric
NetworkX	Hagberg et al., 2008	https://networkx.org/
SweetTalk	Bojar et al., 2021	https://github.com/midas-wyss/sweettalk
SweetNet	This paper	https://github.com/BojarLab/SweetNet

## Highlights

- Graph convolutional neural networks are suited for analyzing glycans
- Learned glycan representations cluster species according to their similarity
- Glycan-focused machine learning can identify viral receptors
