# Supplementary material for: Using graph convolutional neural networks to learn a representation for glycans
Source: Cell Rep. Author manuscript; Available in PMC 2022 Jun 20. (PMC9208909; doi:10.1016/j.celrep.2021.109251)
Supplement: Document S1. Figures S1–S4 [file NIHMS1816564-supplement-Document_S1__Figures_S1_S4.pdf]

Cell Reports, Volume *35*

Supplemental information

Using graph convolutional neural networks to learn a representation for glycans

Rebekka Burkholz, John Quackenbush, and Daniel Bojar

Using Graph Convolutional Neural Networks to Learn a Representation for Glycans

Rebekka Burkholz[1], John Quackenbush[2], and Daniel Bojar[3,4,*]

[1]Department of Biostatistics, Harvard School of Public Health, Boston, MA, USA

[2]Department of Biostatistics, Harvard School of Public Health, Boston, MA, USA; Channing Division of Network Medicine, Department of Medicine, Brigham and Women's Hospital and Harvard Medical School, Boston, MA, USA; Department of Biostatistics and Computational Biology, Dana-Farber Cancer Institute, Boston, MA, USA.

[3]Department of Chemistry and Molecular Biology, University of Gothenburg, Gothenburg, Sweden. Wallenberg Centre for Molecular and Translational Medicine, University of Gothenburg, Gothenburg, Sweden.

[4]Lead Contact

[*]Corresponding author: daniel.bojar@gu.se

Supplementary Figures

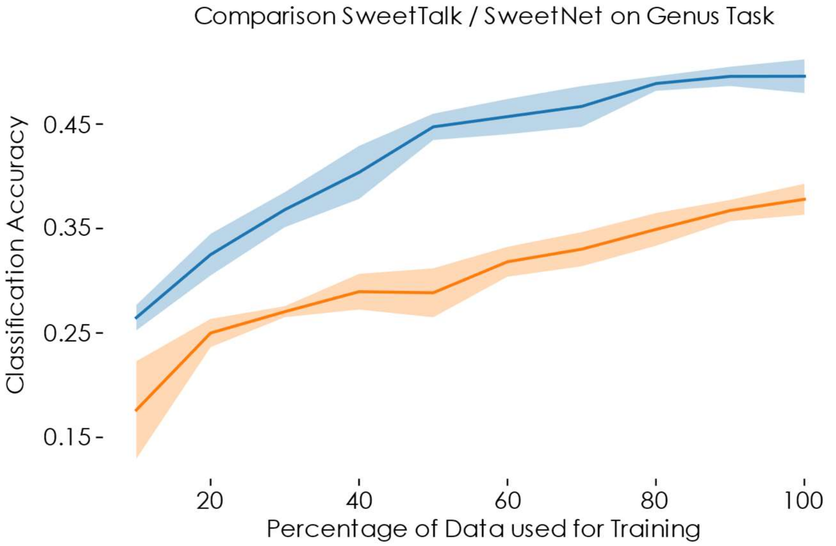

Figure S1. Model comparison regarding data efficiency; Related to Table 1. SweetNet-based (blue) and SweetTalk-based (orange) models were trained to predict the genus a given glycan sequence stemmed from. We trained these models with a random selection of 10-100% of the training dataset and evaluated the resulting model on a separate validation set comprising 20% of the overall data prior to selecting the training data. The data shown are the means (line) and standard deviations (shaded area) of five training runs (N = 5) for each percentage of the training data we tested.

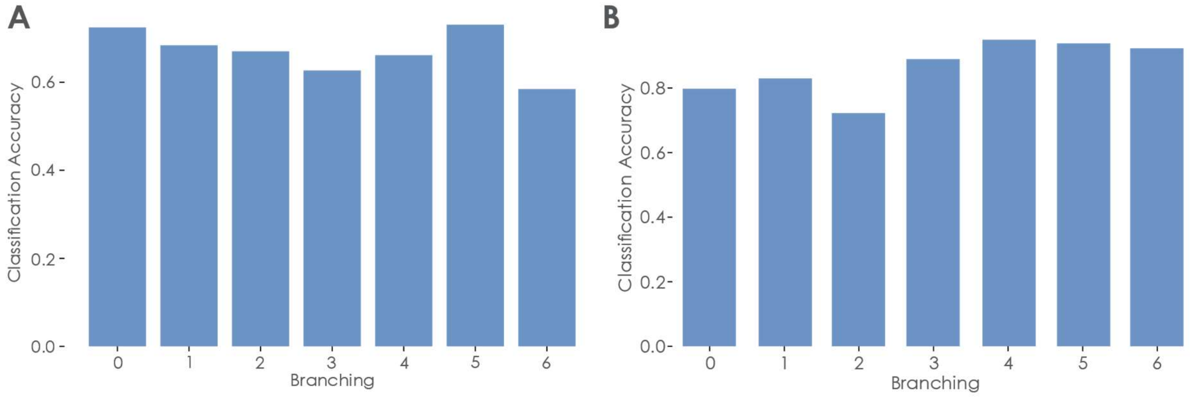

Figure S2. The influence of branching on model performance; Related to Table 1. (A-B) Assessing model performance at every level of branching. For the trained SweetTalk-based (**A**) and SweetNet-based (**B**) models to predict the taxonomic class of a glycan, we depict the averaged prediction accuracy on the entire dataset for every level of glycan branching up to a branching of six, as higher levels of branching were too rare in our dataset for firm conclusions.

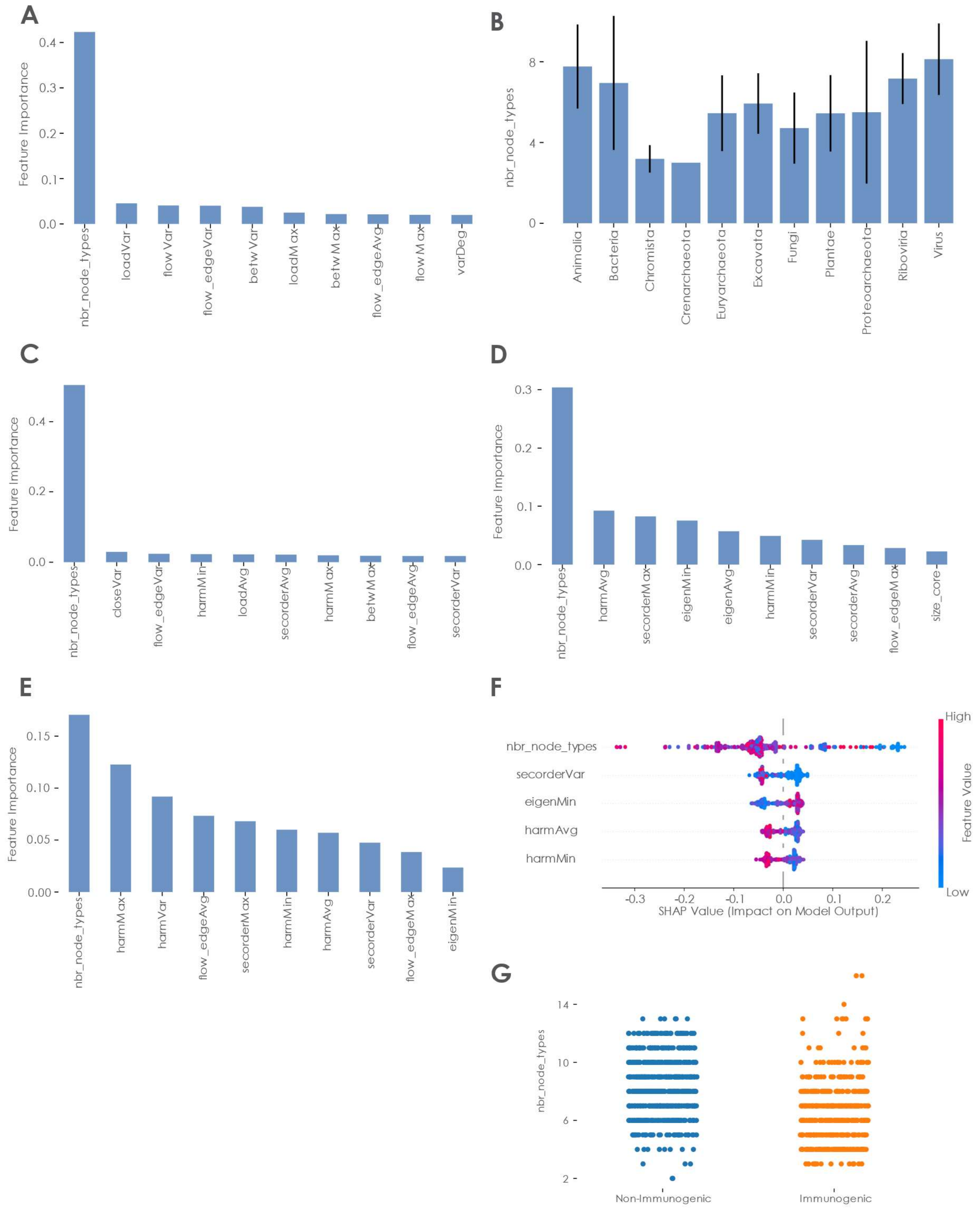

Figure S3. Graph properties important for glycan classification; Related to Figure 2. (A) Feature importance for a Random Forest model trained to predict the taxonomic kingdom of a glycan. The 10 most important features are shown. (**B**) Distribution of nbr_node_types across taxonomic kingdoms. Means and standard deviations of nbr_node_types for each taxonomic kingdom are shown. (**C-E)** Feature importance for a Random Forest model trained to predict the contribution of a glycan to pathogenicity (**C**), glycan immunogenicity (**D**), and glycan class (**E**). The 10 most important features are shown. (**F)** SHAP (SHapley Additive exPlanations) values for important variables of the glycan immunogenicity prediction model. The effect of variable values on the prediction is shown. (**G)** Distribution of number of node types between non-immunogenic and immunogenic glycans. Using a Welch's t-test, we ascertained that the difference is highly significant (p-value: 1.47e-45).

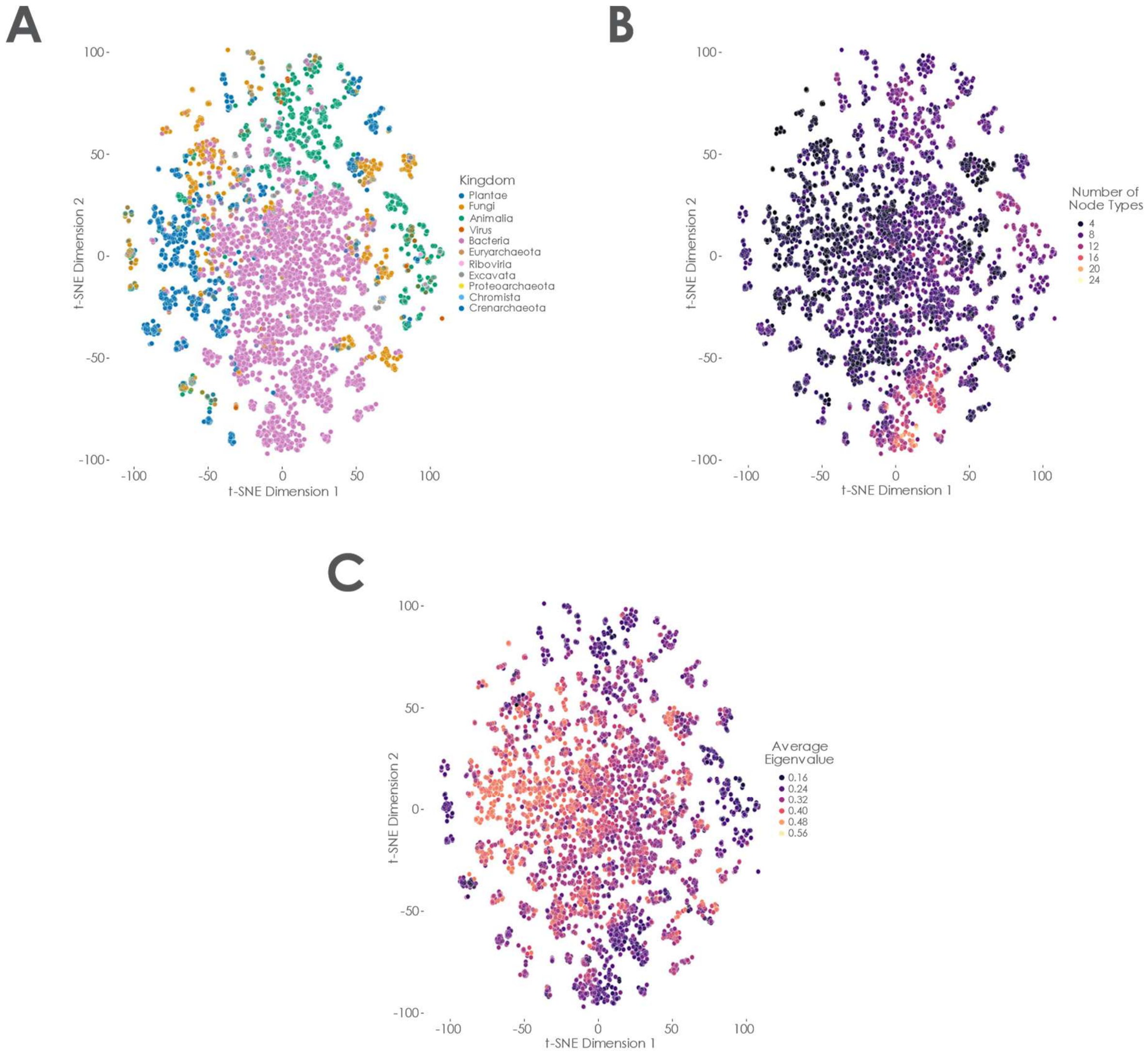

Figure S4. Clusters of glycans with certain graph properties in SweetNet representations; Related to Figure 2. (A-C) Glycan representations for all glycans with taxonomic information in our dataset were generated by using them as input for our trained kingdom-level SweetNet model and extracting the representation learned by the model, shown here via t-SNE. We then colored glycans according to their taxonomic kingdom (**A**), number of node types (**B**), or average eigenvalues (**C**).
